# Neural Flocking: MPC-Based Supervised Learning of Flocking Controllers

**DOI:** 10.1007/978-3-030-45231-5_1

**Published:** 2020-04-17

**Authors:** Usama Mehmood, Shouvik Roy, Radu Grosu, Scott A. Smolka, Scott D. Stoller, Ashish Tiwari

**Affiliations:** 8 ENS/CNRS, Université Paris-Saclay, Cachan, France; 9 University of Duisburg-Essen, Duisburg, Germany; 10 Stony Brook University, Stony Brook, NY USA; 11 Technische Universitat Wien, Wien, Austria; 12 Microsoft Research, San Francisco, CA USA

**Keywords:** Flocking, Model Predictive Control, Distributed Neural Controller, Deep Neural Network, Supervised Learning

## Abstract

We show how a symmetric and fully distributed flocking controller can be synthesized using Deep Learning from a centralized flocking controller. Our approach is based on *Supervised Learning*, with the centralized controller providing the training data, in the form of trajectories of state-action pairs. We use Model Predictive Control (MPC) for the centralized controller, an approach that we have successfully demonstrated on flocking problems. MPC-based flocking controllers are high-performing but also computationally expensive. By learning a symmetric and distributed neural flocking controller from a centralized MPC-based one, we achieve the best of both worlds: the neural controllers have high performance (on par with the MPC controllers) and high efficiency. Our experimental results demonstrate the sophisticated nature of the distributed controllers we learn. In particular, the neural controllers are capable of achieving myriad flocking-oriented control objectives, including flocking formation, collision avoidance, obstacle avoidance, predator avoidance, and target seeking. Moreover, they generalize the behavior seen in the training data to achieve these objectives in a significantly broader range of scenarios. In terms of verification of our neural flocking controller, we use a form of statistical model checking to compute confidence intervals for its convergence rate and time to convergence.

### 1 Introduction

With the introduction of Reynolds rule-based model [16, 17], it is now possible to understand the flocking problem as one of distributed control. Specifically, in this model, at each time-step, each agent executes a control law given in terms of the weighted sum of three competing forces to determine its next acceleration. Each of these forces has its own rule: *separation* (keep a safe distance away from your neighbors), *cohesion* (move towards the centroid of your neighbors), and *alignment* (steer toward the average heading of your neighbors). Reynolds

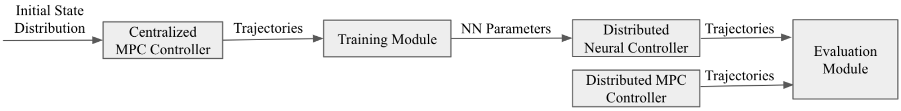

Fig. 1: Neural Flocking Architecture controller is *distributed*; i.e., it is executed separately by each agent, using information about only itself and nearby agents, and without communication. Furthermore, it is *symmetric*; i.e., every agent runs the same controller (same code).

We subsequently showed that a simpler, more declarative approach to the flocking problem is possible [11]. In this setting, flocking is achieved when the agents combine to minimize a system-wide *cost function*. We presented centralized and distributed solutions for achieving this form of "declarative flocking" (DF), both of which were formulated in terms of Model-Predictive Control (MPC) [2].

Another advantage of DF over the ruled-based approach exemplified by Reynolds model is that it allows one to consider additional control objectives (e.g., obstacle and predator avoidance) simply by extending the cost function with additional terms for these objectives. Moreover, these additional terms are typically quite straightforward in nature. In contrast, deriving behavioral rules that achieve the new control objectives can be a much more challenging task.

An issue with MPC is that computing the next control action can be computationally expensive, as MPC searches for an action sequence that minimizes the cost function over a given prediction horizon. This renders MPC unsuitable for real-time applications with short control periods, for which flocking is a prime example. Another potential problem with MPC-based approaches to flocking is its performance (in terms of achieving the desired flight formation), which may suffer in a fully distributed setting.

In this paper, we present *Neural Flocking* (NF), a new approach to the flocking problem that uses Supervised Learning to learn a symmetric and fully distributed flocking controller from a centralized MPC-based controller. By doing so, we achieve the best of both worlds: high performance (on par with the MPC controllers) in terms of meeting flocking flight-formation objectives, and high efficiency leading to real-time flight controllers. Moreover, our NF controllers can easily be parallelized on hardware accelerators such as GPUs and TPUs.

Figure 1 gives an overview of the NF approach. A high-performing centralized MPC controller provides the labeled training data to the learning agent: a symmetric and distributed neural controller in the form of a deep neural network (DNN). The training data consists of trajectories of state-action pairs, where a state contains the information known to an agent at a time step (e.g., its own position and velocity, and the position and velocity of its neighbors), and the action (the label) is the acceleration assigned to that agent at that time step by the centralized MPC controller.

We formulate and evaluate NF in a number of essential flocking scenarios: basic flocking with inter-agent collision avoidance, as in [11], and more advanced scenarios with additional objectives, including obstacle avoidance, predator avoidance, and target seeking by the flock. We conduct an extensive performance evaluation of NF. Our experimental results demonstrate the sophisticated nature of NF controllers. In particular, they are capable of achieving all of the stated control objectives. Moreover, they generalize the behavior seen in the training data in order to achieve these objectives in a significantly broader range of scenarios. In terms of verification of our neural controller, we use a form of statistical model checking [5, 10] to compute confidence intervals for its rate of convergence to a flock and for its time to convergence.

### 2 Background

We consider a set of $n$ dynamic agents $\mathcal{A} = \{1, \ldots, n\}$ that move according to the following discrete-time equations of motion:

$$\begin{aligned} p_i(k+1) &= p_i(k) + dt \cdot v_i(k), \quad |v_i(k)| < \bar{v} \\ v_i(k+1) &= v_i(k) + dt \cdot a_i(k), \quad |a_i(k)| < \bar{a} \end{aligned} \tag{1}$$

where $p_i(k) \in \mathbb{R}^2$, $v_i(k) \in \mathbb{R}^2$, $a_i(k) \in \mathbb{R}^2$ are the position, velocity and acceleration of agent $i \in \mathcal{A}$ respectively at time step $k$, and $dt \in \mathbb{R}^+$ is the time step. The magnitudes of velocities and accelerations are bounded by $\bar{v}$ and $\bar{a}$, respectively. Acceleration $a_i(k)$ is the control input for agent $i$ at time step $k$. The acceleration is updated after every $\eta$ time steps i.e., $\eta \cdot dt$ is the control period. The flock *configuration* at time step $k$ is thus given by the following vectors (in boldface):

$$\mathbf{p}(k) = [p_1^T(k) \cdots p_n^T(k)]^T \tag{2}$$

$$\mathbf{v}(k) = [v_1^T(k) \cdots v_n^T(k)]^T \tag{3}$$

$$\mathbf{a}(k) = [a_1^T(k) \cdots a_n^T(k)]^T \tag{4}$$

The configuration vectors are referred to without the time indexing as $\mathbf{p}$, $\mathbf{v}$, and $\mathbf{a}$. The *neighborhood* of agent $i$ at time step $k$, denoted by $\mathcal{N}_i(k) \subseteq \mathcal{A}$, contains its $\mathcal{N}$-nearest neighbors, i.e., the $\mathcal{N}$ other agents closest to it. We use this definition (in Section 2.2 to define a distributed-flocking cost function) for simplicity, and expect that a radius-based definition of neighborhood would lead to similar results for our distributed flocking controllers.

#### 2.1 Model-Predictive Control

Model-Predictive control (MPC) [2] is a well-known control technique that has recently been applied to the flocking problem [11, 19, 20]. At each control step, an optimization problem is solved to find the optimal sequence of control actions (agent accelerations in our case) that minimizes a given cost function with respect to a predictive model of the system. The first control action of the optimal control sequence is then applied to the system; the rest is discarded. In the computation of the cost function, the predictive model is evaluated for a finite prediction horizon of $T$ control steps.

MPC-based flocking models can be categorized as *centralized* or *distributed.* A *centralized* model assumes that complete information about the flock is available to a single "global" controller, which uses the states of all agents to compute their next optimal accelerations. The following optimization problem is solved by a centralized MPC controller at each control step $k$:

$$\min_{\mathbf{a}(k|k),\ldots,\mathbf{a}(k+T-1|k) < \bar{a}} \quad J(k) + \lambda \cdot \sum_{t=0}^{T-1} \|\mathbf{a}(k+t \mid k)\|^2 \tag{5}$$

The first term $J(k)$ is the centralized model-specific cost, evaluated for $T$ control steps (this embodies the predictive aspect of MPC), starting at time step $k$. It encodes the control objective of minimizing the cost function $J(k)$. The second term, scaled by a weight $\lambda > 0$, penalizes large control inputs: $\mathbf{a}(k+t \mid k)$ are the predictions made at time step $k$ for the accelerations at time step $k+t$.

In *distributed MPC*, each agent computes its acceleration based only on its own state and its local knowledge, e.g., information about its neighbors:

$$\min_{a_i(k|k),\ldots,a_i(k+T-1|k) < \bar{a}} \quad J_i(k) + \lambda \cdot \sum_{t=0}^{T-1} \|a_i(k+t \mid k)\|^2 \tag{6}$$

$J_i(k)$ is the distributed, model-specific cost function for agent $i$, analogous to $J(k)$. In a distributed setting where an agent's knowledge of its neighbors' behavior is limited, an agent cannot calculate the exact future behavior of its neighbors. Hence, the predictive aspect of $J_i(k)$ must rely on some assumption about that behavior during the prediction horizon. Our distributed cost functions are based on the assumption that the neighbors have zero accelerations during the prediction horizon. While this simple design is clearly not completely accurate, our experiments show that it still achieves good results.

### 2.2 Declarative Flocking

Declarative flocking (DF) is a high-level approach to designing flocking algorithms based on defining a suitable cost function for MPC [11]. This is in contrast to the operational approach, where a set of rules are used to capture flocking behavior, as in Reynolds model. For basic flocking, the DF cost function contains two terms: (1) a *cohesion* term based on the squared distance between each pair of agents in the flock; and (2) a *separation* term based on the inverse of the squared distance between each pair of agents. The flock evolves toward a configuration in which these two opposing forces are balanced. The cost function $J^C$ for centralized DF, i.e., centralized MPC (CMPC), is as follows:

$$J^C(\mathbf{p}) = \frac{2}{|\mathcal{A}| \cdot (|\mathcal{A}|-1)} \cdot \sum_{i \in \mathcal{A}} \sum_{j \in \mathcal{A}, i<j} \|p_{ij}\|^2 + \omega_s \cdot \frac{1}{\|p_{ij}\|^2} \tag{7}$$

where $\omega_s$ is the weight of the separation term and controls the density of the flock. The cost function is normalized by the number of pairs of agents, $\frac{|\mathcal{A}|\cdot(|\mathcal{A}-1|)}{2}$; as such, the cost does not depend on the size of the flock. The control law for CMPC is given by Eq. (5), with $J(k) = \sum_{t=1}^{T} J^{\mathrm{C}}\left(\mathbf{p}(k+t \mid k)\right)$.

The basic flocking cost function for distributed DF is similar to that for CMPC, except that the cost function $J_i^D$ for agent $i$ is computed over its set of neighbors $\mathcal{N}_i(k)$ at time $k$:

$$J_i^{\mathrm{D}}\left(\mathbf{p}(k)\right) = \frac{1}{|\mathcal{N}_i(k)|} \cdot \sum_{j \in \mathcal{N}_i(k)} \|p_{ij}\|^2 + \omega_s \cdot \sum_{j \in \mathcal{N}_i(k)} \frac{1}{\|p_{ij}\|^2} \tag{8}$$

The control law for agent $i$ is given by Eq. (6), with $J_i(k) = \sum_{t=1}^{T} J_i^{\mathrm{D}}\left(\mathbf{p}(k+t \mid k)\right)$.

### 3 Additional Control Objectives

The cost functions for basic flocking given in Eqs. (7) and (8) are designed to ensure that in the steady state, the agents are well-separated. Additional goals such as obstacle avoidance, predator avoidance, and target seeking are added to the MPC formulation as weighted cost-function terms. Different objectives can be combined by including the corresponding terms in the cost function as a weighted sum.

*Cost-Function Term for Obstacle Avoidance.* We consider multiple rectangular obstacles which are distributed randomly in the field. For a set of $m$ rectangular obstacles $\mathcal{O} = \{\mathcal{O}_1, \mathcal{O}_2, ..., \mathcal{O}_m\}$, we define the cost function term for obstacle avoidance as:

$$J_{OA}(\mathbf{p}, \mathbf{o}) = \frac{1}{|\mathcal{A}||\mathcal{O}|} \sum_{i \epsilon \mathcal{A}} \sum_{j \epsilon \mathcal{O}} \frac{1}{\left\| p_i - o_j^{(i)} \right\|^2} \tag{9}$$

where $\mathbf{o}$ is the set of points on the obstacle boundaries and $o_j^{(i)}$ is the point on the obstacle boundary of the $j^{th}$ obstacle $\mathcal{O}_j$ that is closest to the $i^{th}$ agent.

*Cost-Function Term for Target Seeking.* This term is the average of the squared distance between the agents and the target. Let $g$ denote the position of the fixed target. Then the target-seeking term is as defined as

$$J_{TS}(\mathbf{p}) = \frac{1}{|\mathcal{A}|} \sum_{i \in \mathcal{A}} \|p_i - g\|^2 \tag{10}$$

*Cost-Function Term for Predator Avoidance.* We introduce a single predator, which is more agile than the flocking agents: its maximum speed and acceleration are a factor of $f_p$ greater than $\bar{v}$ and $\bar{a}$, respectively, with $f_p > 1$. Apart from being more agile, the predator has the same dynamics as the agents, given by

Eq. (1). The control law for the predator consists of a single term that causes it to move toward the centroid of the flock with maximum acceleration.

For a flock of $n$ agents and one predator, the cost-function term for predator avoidance is the average of the inverse of the cube of the distances between the predator and the agents. It is given by:

$$J_{PA}\left(\mathbf{p}, p_{pred}\right) = \frac{1}{|\mathcal{A}|} \sum_{i \epsilon \mathcal{A}} \frac{1}{||p_i - p_{pred}||^3} \tag{11}$$

where $p_{pred}$ is the position of the predator. In contrast to the separation term in Eqs. (5)-(6), which we designed to ensure inter-agent collision avoidance, the predator-avoidance term has a cube instead of a square in the denominator. This is to reduce the influence of the predator on the flock when the predator is far away from the flock.

*NF Cost-Function Terms.* The MPC cost functions used in our examination of Neural Flocking are weighted sums of the cost function terms introduced above. We refer to the first term of our centralized DF cost function $J^C(\mathbf{p})$ (see Eq. (7)) as $J_{cohes}(\mathbf{p})$ and the second as $J_{sep}(\mathbf{p})$. We use the following cost functions $J_1$, $J_2$, and $J_3$ for basic flocking with collision avoidance, obstacle avoidance with target seeking, and predator avoidance, respectively.

$$J_1(\mathbf{p}) = J_{cohes}(\mathbf{p}) + \omega_s \cdot J_{sep}(\mathbf{p}) \tag{12a}$$

$$J_2(\mathbf{p}, \mathbf{o}) = J_{cohes}(\mathbf{p}) + \omega_s \cdot J_{sep}(\mathbf{p}) + \omega_o \cdot J_{OA}(\mathbf{p}, \mathbf{o}) + \omega_t \cdot J_{TS}(\mathbf{p}) \tag{12b}$$

$$J_3(\mathbf{p}, p_{pred}) = J_{cohes}(\mathbf{p}) + \omega_s \cdot J_{sep}(\mathbf{p}) + \omega_p \cdot J_{PA}(\mathbf{p}, p_{pred}) \tag{12c}$$

where $\omega_s$ is the weight of the separation term, $\omega_o$ is the weight of the obstacle avoidance term, $\omega_t$ is the weight of the target-seeking term, and $\omega_p$ is the weight of the predator-avoidance term. Note that $J_1$ is equivalent to $J^C$ (Eq. (7)). The weight $\omega_s$ of the separation term is experimentally chosen to ensure that the distance between agents, throughout the simulation, is at least $d_{min}$, the minimum inter-agent distance representing collision avoidance. Similar considerations were given to the choice of values for $\omega_o$ and $\omega_p$. The specific values we used for the weights are: $\omega_s = 2000$, $\omega_o = 1500$, $\omega_t = 10$, and $\omega_p = 500$.

We experimented with an alternative strategy for introducing inter-agent collision avoidance, obstacle avoidance, and predator avoidance into the MPC problem, namely, as *constraints* of the form $d_{min} - p_{ij} < 0$, $d_{min} - ||p_i - o_j^{(i)}|| < 0$, and $d_{min} - ||p_i - p_{pred}|| < 0$, respectively. Using the theory of exact penalty functions [12], we recast the constrained MPC problem as an equivalent unconstrained MPC problem by converting the constraints into a weighted *penalty term*, which is then added to the MPC cost function. This approach rendered the optimization problem difficult to solve due to the non-smoothness of the penalty term. As a result, constraint violations in the form of collisions were observed during simulation.

## 4 Neural Flocking

We learn a *distributed neural controller* (DNC) for the flocking problem using training data in the form of trajectories of state-action pairs produced by a CMPC controller. In addition to basic flocking with inter-agent collision avoidance, the DNC exhibits a number of other flocking-related behaviors, including obstacle avoidance, target seeking, and predator avoidance. We also show how the learned behavior exhibited by the DNC generalizes over a larger number of agents than what was used during training to achieve successful collision-free flocking in significantly larger flocks.

We use *Supervised Learning* to train the DNC. Supervised Learning learns a function that maps an input to an output based on example sequences of input-output pairs. In our case, the trajectory data obtained from CMPC contains both the training inputs and corresponding labels (outputs): the state of an agent in the flock (and that of its nearest neighbors) at a particular time step is the input, and that agent's acceleration at the same time step is the label.

### 4.1 Training Distributed Flocking Controllers

We use Deep Learning to synthesize a distributed and symmetric neural controller from the training data provided by the CMPC controller. Our objective is to learn basic flocking, obstacle avoidance with target seeking, and predator avoidance. Their respective CMPC-based cost functions are given in Sections 2.2 and 3. All of these control objectives implicitly also include inter-agent collision avoidance by virtue of the separation term in Eq. 7.

For each of these control objectives, DNC training data is obtained from CMPC trajectory data generated for $n = 15$ agents, starting from initial configurations in which agent positions and velocities are uniformly sampled from $[-15, 15]^2$ and $[0, 1]^2$, respectively. All training trajectories are 1,000 time steps in duration.

We further ensure that the initial configurations are *recoverable*; i.e., no two agents are so close to each other that they cannot avoid a collision by resorting to maximal accelerations. We learn a single DNC from the state-action pairs of all $n$ agents. This yields a symmetric distributed controller, which we use for each agent in the flock during evaluation.

*Basic Flocking.* Trajectory data for basic flocking is generated using the cost function given in Eq. (7). We generate 200 trajectories, each of which (as noted above) is 1,000 time steps long. The input to the NN is the position and velocity of each agent along with the positions and velocities of its $\mathcal{N}$-nearest neighbors. This yields $200 \cdot 1,000 \cdot 15 = 3M$ total training samples.

Let us refer to the agent (the DNC) being learned as $\mathcal{A}_0$. Since we use neighborhood size $\mathcal{N} = 14$, the input to the NN is of the form $[p_0^x\ p_0^y\ v_0^x\ v_0^y\ p_1^x\ p_1^y\ v_1^x\ v_1^y\ \dots\ p_{14}^x\ p_{14}^y\ v_{14}^x\ v_{14}^y]$, where $p_0^x$, $p_0^y$ are the position coordinates and $v_0^x$, $v_0^y$ velocity coordinates for agent $\mathcal{A}_0$, and $p_{1\dots14}^x$, $p_{1\dots14}^y$ and $v_{1\dots14}^x$, $v_{1\dots14}^y$ are the position and velocity vectors of its neighbors. Since this input vector has 60 components, the input to the NN consists of 60 features.

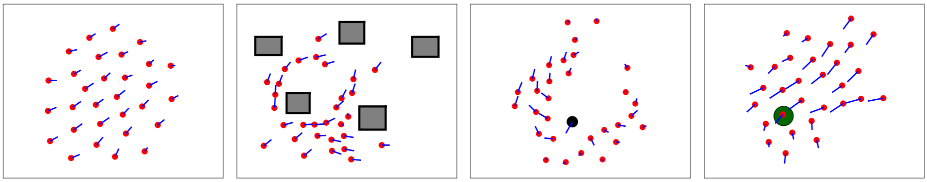

(a) Basic flocking (b) Obstacle avoid. (c) Predator avoid. (d) Target seeking

Fig. 2: Snapshots of DNC flocking behaviors for 30 agents

*Obstacle Avoidance with Target Seeking.* For obstacle avoidance with target seeking, we use CMPC with the cost function given in Eq. (12b). The target is located beyond the obstacles, forcing the agents to move through the obstacle field. For the training data, we generate 100 trajectories over 4 different obstacle fields (25 trajectories per obstacle field). The input to the NN consists of the 92 features $[p_0^x\ p_0^y\ v_0^x\ v_0^y\ o_0^x\ o_0^y \ldots p_{14}^x\ p_{14}^y\ v_{14}^x\ v_{14}^y\ o_{14}^x\ o_{14}^y\ g^x\ g^y]$, where $o_0^x$, $o_0^y$ is the closest point on any obstacle to agent $\mathcal{A}_0$; $o_{1\ldots14}^x$ , $o_{1\ldots14}^y$ give the closest point on any obstacle for the 14 neighboring agents, and $g^x$, $g^y$ is the target location.

*Predator Avoidance.* The CMPC cost function for predator avoidance is given in Eq. (12c). The position, velocity, and the acceleration of the predator are denoted by $p_{pred}$, $v_{pred}$, $a_{pred}$, respectively. We take $f_p = 1.40$; hence $\bar{v}_{pred} = 1.40\,\bar{v}$ and $\bar{a}_{pred} = 1.40\,\bar{a}$. The input features to the NN are the positions and velocities of agent $\mathcal{A}_0$ and its $\mathcal{N}$-nearest neighbors, and the position and velocity of the predator. The input with 64 features thus has the form $[p_0^x\ p_0^y\ v_0^x\ v_0^y\ \ldots\ p_{14}^x\ p_{14}^y\ v_{14}^x\ v_{14}^y\ p_{pred}^x\ p_{pred}^y\ v_{pred}^x\ v_{pred}^y]$.

## 5 Experimental Evaluation

This section contains the results of our extensive performance analysis of the distributed neural flocking controller (DNC), taking into account various control objectives: basic flocking with collision avoidance, obstacle avoidance with target seeking, and predator avoidance. As illustrated in Fig. 1, this involves running CMPC to generate the training data for the DNCs, whose performance we then compare to that of the DMPC and CMPC controllers. We also show that the DNC flocking controllers generalize the behavior seen in the training data to achieve successful collision-free flocking in flocks significantly larger in size than those used during training. Finally, we use Statistical Model Checking to obtain confidence intervals for DNC's correctness/performance.

### 5.1 Preliminaries

The CMPC and DMPC control problems defined in Section 2.1 are solved using MATLAB fmincon optimizer. In the training phase, the size of the flock is

$n = 15$. For obstacle-avoidance with target-seeking, we use 5 obstacles with the target located at [60,50]. The simulation time is 100, $dt = 0.1$ time units, and $\eta = 3$, where (recall) $\eta \cdot dt$ is the control period. Further, the agent velocity and acceleration bounds are $\bar{v} = 2.0$ and $\bar{a} = 1.5$.

We use $d_{min} = 1.5$ as the minimum inter-agent distance for collision avoidance, $d^{obs}_{min} = 1$ as the minimum agent-obstacle distance for obstacle avoidance, and $d^{pred}_{min} = 1.5$ as the minimum agent-predator distance for predator avoidance. For initial configurations, recall that agent positions and velocities are uniformly sampled from $[-15, 15]^2$ and $[0, 1]^2$, respectively, and we ensure that they are *recoverable*; i.e., no two agents are so close to each other that they cannot avoid a collision when resorting to maximal accelerations. The predator starts at rest from a fixed location at a distance of 40 from the flock center.

For training, we considered 15 agents and 200 trajectories per agent, each trajectory 1,000 time steps in length. This yielded a total of 3,000,000 training samples. Our neural controller is a fully connected feed-forward Deep Neural Network (DNN), with 5 hidden layers, 84 neurons per hidden layer, and with a ReLU activation function. We use an iterative approach for choosing the DNN hyperparameters and architecture where we continuously improve our NN, until we observe satisfactory performance by the DNC.

For training the DNNs, we use Keras [3], which is a high-level neural network API written in Python and capable of running on top of TensorFlow. To generate the NN model, Keras uses the Adam optimizer [8] with the following settings: $lr = 10^{-2}$, $\beta_1 = 0.9$, $\beta_2 = 0.999$, $\epsilon = 10^{-8}$. The batch size (number of samples processed before the model is updated) is 2,000, and the number of epochs (number of complete passes through the training dataset) used for training is 1,000. For measuring training loss, we use the mean-squared error metric.

For basic flocking, DNN input vectors have 60 features and the number of trainable DNN parameters is 33,854. For flocking with obstacle-avoidance and target-seeking, input vectors have 92 features and the number of trainable parameters is 36,542. Finally, for flocking with predator-avoidance, input vectors have 64 features and the resulting number of trainable DNN parameters is 34,190.

To test the trained DNC, we generated 100 simulations (runs) for each of the desired control objectives: basic flocking with collision avoidance, flocking with obstacle avoidance and target seeking, and flocking with predator avoidance. The results presented in Tables 1, were obtained using the same number of agents and obstacles and the same predator as in the training phase. We also ran tests that show DNC controllers can achieve collision-free flocking with obstacle avoidance where the numbers of agents and obstacles are greater than those used during training.

#### 5.2 Results for Basic Flocking

We use flock diameter, inter-agent collision count and velocity convergence [20] as performance metrics for flocking behavior. At any time step, the *flock diameter* $D(\mathbf{p}) = \max_{(i,j) \in \mathcal{A}} \|p_{ij}\|$ is the largest distance between any two agents in the flock. We calculate the average converged diameter by averaging the flock diameter

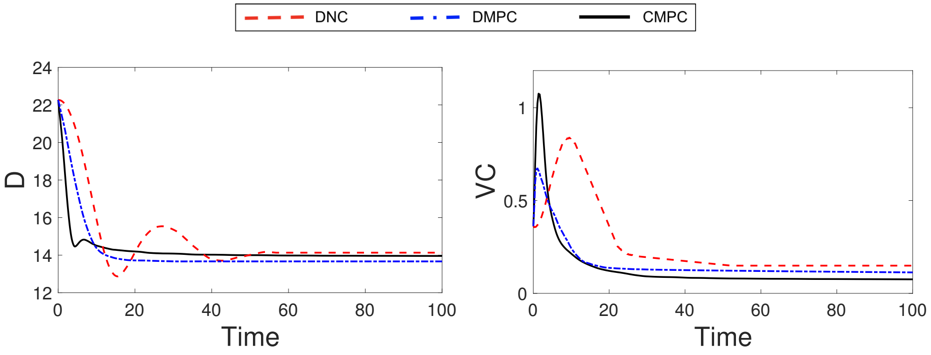

(a) Flock diameter (b) Velocity convergence

Fig. 3: Performance comparison for basic flocking with collision avoidance, averaged over 100 test runs.

in the final time step of the simulation over the 100 runs. An inter-agent collision (IC) occurs when the distance between two agents at any point in time is less than $d_{min}$. The IC rate (ICR) is the average number of ICs per test-trajectory time-step. The velocity convergence $VC(\mathbf{v}) = (1/n)\left(\sum_{i\in\mathcal{A}} \|v_i - (\sum_{j=1}^{n} v_j)/n\|^2\right)$ is the average of the squared magnitude of the discrepancy between the velocities of agents and the flock's average velocity. For all the metrics, lower values are better, indicating a denser and more coherent flock with fewer collisions. A successful flocking controller should also ensure that values of $D(\mathbf{p})$ and $VC(\mathbf{v})$ eventually stabilize.

Fig. 3 and Table 1 compare the performance of the DNC on the basic-flocking problem for 15 agents to that of the MPC controllers. Although the DMPC and CMPC outperform the DNC, the difference is marginal. An important advantage of the DNC over DMPC is that they are much faster. Executing a DNC controller requires a modest number of arithmetic operations, whereas executing an MPC controller requires simulation of a model and controller over the prediction horizon. In our experiments, on average, the CMPC takes 1209 msec of CPU time for the entire flock and DMPC takes 58 msec of CPU time per agent, whereas the DNC takes only 1.6 msec.

Table 1: Performance comparison for BF with 15 agents on 100 test runs

| | Avg. Conv. Diameter | ICR | Velocity Convergence |
|---|---|---|---|
| DNC | 14.13 | 0 | 0.15 |
| DMPC | 13.67 | 0 | 0.11 |
| CMPC | 13.84 | 0 | 0.10 |

Table 2: DNC Performance Generalization for BF

| Agents | Avg. Conv. Diameter | Conv. Rate (%) | Avg. Conv. Time | ICR |
|---|---|---|---|---|
| 15 | 14.13 | 100 | 52.15 | 0 |
| 20 | 16.45 | 97 | 58.76 | 0 |
| 25 | 19.81 | 94 | 64.11 | 0 |
| 30 | 23.24 | 92 | 72.08 | 0 |
| 35 | 30.57 | 86 | 83.84 | 0.008 |
| 40 | 38.66 | 81 | 95.32 | 0.019 |

#### 5.3 Results for Obstacle and Predator Avoidance

For obstacle and predator avoidance, collision rates are used as a performance metric. An obstacle-agent collision (OC) occurs when the distance between an agent and the closest point on any obstacle is less than $d^{obs}_{min}$. A predator-agent collision (PC) occurs when the distance between an agent and the predator is less than $d^{pred}_{min}$. The OC rate (OCR) is the average number of OCs per test-trajectory time-step, and the PC rate (PCR) is defined similarly. Our test results show that the DNC, along with the DMPC and CMPC, is collision-free (i.e., each of ICR, OCR, and PCR is zero) for 15 agents, with the exception of DMPC for predator avoidance where PCR $= 0.013$. We also observed that the flock successfully reaches the target location in all 100 test runs.

#### 5.4 DNC Generalization Results

Tables 2–3 present DNC generalization results for basic flocking (BF), obstacle avoidance (OA), and predator avoidance (PA), with the number of agents ranging from 15 (the flock size during training) to 40. In all of these experiments, we use a neighborhood size of $\mathcal{N} = 14$, the same as during training. Each controller was evaluated with 100 test runs. The performance metrics in Table 2 are the average converged diameter, convergence rate, average convergence time, and ICR.

The convergence rate is the fraction of successful flocks over 100 runs. The collection of agents is said to have converged to a flock (with collision avoidance) if the value of the global cost function is less than the convergence threshold. We use a convergence threshold of $J_1(\mathbf{p}) \leq 150$, which was chosen based on its proximity to the value achieved by CMPC. We use the cost function from Eq. 12a to calculate our success rate because we are showing convergence rate for basic flocking. The average convergence time is the time when the global cost function first drops below the success threshold and remains below it for the rest of the run, averaged over all 100 runs. Even with a local neighborhood of size 14, the results demonstrate that the DNC can successfully generalize to a large number of agents for all of our control objectives.

Table 3: DNC Generalization Performance for OA and PA

| | OA | | PA | |
|---|---|---|---|---|
| Agents | ICR | OCR | ICR | PCR |
| 15 | 0 | 0 | 0 | 0 |
| 20 | 0 | 0 | 0 | 0 |
| 25 | 0 | 0 | 0 | 0 |
| 30 | 0 | 0 | 0 | 0 |
| 35 | 0.011 | 0.009 | 0.013 | 0.010 |
| 40 | 0.021 | 0.018 | 0.029 | 0.023 |

#### 5.5 Statistical Model Checking Results

We use Monte Carlo (MC) approximation as a form of Statistical Model Checking [5, 10] to compute confidence intervals for the DNC's convergence rate to a flock with collision avoidance and for the (normalized) convergence time. The convergence rate is the fraction of successful flocks over $N$ runs. The collection of agent is said to have converged to a successful flock with collision avoidance if the global cost function $J_1(\mathbf{p}) \leq 150$, where $J_1(\mathbf{p})$ is cost function for basic flocking defined in Eq. 12a.

The main idea of MC is to use $N$ random variables, $Z_1, \ldots, Z_N$, also called samples, IID distributed according to a random variable $Z$ with mean $\mu_Z$, and to take the sum $\tilde{\mu}_Z = (Z_1 + \ldots + Z_N)/N$ as the value approximating the mean $\mu_Z$. Since an exact computation of $\mu_Z$ is almost always intractable, an MC approach is used to compute an $(\epsilon, \delta)$-approximation of this quantity.

*Additive Approximation* [6] is an $(\epsilon, \delta)$-approximation scheme where the mean $\mu_Z$ of an RV $Z$ is approximated with absolute error $\epsilon$ and probability $1 - \delta$:

$$Pr[\mu_Z - \epsilon \leq \tilde{\mu}_Z \leq \mu_Z + \epsilon] \geq 1 - \delta \tag{13}$$

where $\tilde{\mu}_Z$ is an approximation of $\mu_Z$. An important issue is to determine the number of samples $N$ needed to ensure that $\tilde{\mu}_Z$ is an $(\epsilon, \delta)$-approximation of $\mu_Z$. If $Z$ is a Bernoulli variable expected to be large, one can use the Chernoff-Hoeffding instantiation of the Bernstein inequality and take $N$ to be $N = 4\ln(2/\delta)/\epsilon^2$, as in [6]. This results in the *additive approximation algorithm* [5], defined in Algorithm 1.

We use this algorithm to obtain a joint $(\epsilon, \delta)$-approximation of the mean convergence rate and mean normalized convergence time for the DNC. Each sample $Z_i$ is based on the result of an execution obtained by simulating the system starting from a random initial state, and we take $Z = (B, R)$, where $B$ is a Boolean variable indicating whether the agents converged to a flock during the execution, and $R$ is a real value denoting the normalized convergence time. The normalized convergence time is the time when the global cost function first drops below the convergence threshold and remains below it for the rest of the run, measured as a fraction of the total duration of the run. The assumptions

**Algorithm 1: Additive Approximation Algorithm**

**Input:** ($\epsilon$, $\delta$) with $0 < \epsilon < 1$ and $0 < \delta < 1$
**Input:** Random variables $Z_i$, IID
**Output:** $\tilde{\mu}_Z$ approximation of $\mu_Z$
$N = 4\ln(2/\delta)/\epsilon^2$;
**for** *(i=0; $i \leq N$; i++)* **do**
  $S = S + Z_i$;
$\tilde{\mu_Z} = S/N$; **return** $\tilde{\mu}_Z$;

Table 4: SMC results for DNC convergence rate and normalized convergence time; $\epsilon = 0.01$, $\delta = 0.0001$

| Agents | $\tilde{\mu}_{CR}$ | $\tilde{\mu}_{CT}$ |
|---|---|---|
| 15 | 0.99 | 0.53 |
| 20 | 0.97 | 0.58 |
| 25 | 0.94 | 0.65 |
| 30 | 0.91 | 0.71 |
| 35 | 0.86 | 0.84 |
| 40 | 0.80 | 0.95 |

about $Z$ required for validity of the additive approximation hold, because RV $B$ is a Bernoulli variable, the convergence rate is expected to be large (i.e., closer to 1 than to 0), and the proportionality constraint of the Bernstein inequality is also satisfied for RV $R$.

In these experiments, the initial configurations are sampled from the same distributions as in Section 5.1, and we set $\epsilon = 0.01$ and $\delta = 0.0001$, to obtain $N =$ 396,140. We perform the required set of $N$ simulations for 15, 20, 25, 30, 35 and 40 agents. Table 4 presents the results, specifically, the $(\epsilon, \delta)$-approximations $\tilde{\mu}_{CR}$ and $\tilde{\mu}_{CT}$ of the mean convergence rate and the mean normalized convergence time, respectively. While the results for the convergence rate are (as expected) numerically similar to the results in Table 2, the results in Table 4 are much stronger, because they come with the guarantee that they are $(\epsilon, \delta)$-approximations of the actual mean values.

### 6 Related Work

In [18], a flocking controller is synthesized using multi-agent reinforcement learning (MARL) and natural evolution strategies (NES). The target model from which the system learns is Reynolds flocking model [16]. For training purposes, a list of metrics called *entropy* are chosen, which provide a measure of the collective behavior displayed by the target model. As the authors of [18] observe, this technique does not quite work: although it consistently leads to agents forming recognizable patterns during simulation, agents self-organized into a cluster instead of flowing like a flock.

In [9], reinforcement learning and flocking control are combined for the purpose of predator avoidance, where the learning module determines safe spaces in which the flock can navigate to avoid predators. Their approach to predator avoidance, however, isn't distributed as it requires a majority consensus by the flock to determine its action to avoid predators. They also impose an $\alpha$-lattice structure [13] on the flock. In contrast, our approach is geometry-agnostic and achieves predator avoidance in a distributed manner.

In [7], an uncertainty-aware reinforcement learning algorithm is developed to estimate the probability of a mobile robot colliding with an obstacle in an unknown environment. Their approach is based on bootstrap neural networks using dropouts, allowing it to process raw sensory inputs. Similarly, a learning-based approach to robot navigation and obstacle avoidance is presented in [14]. They train a model that maps sensor inputs and the target position to motion commands generated by the ROS [15] navigation package. Our work in contrast considers obstacle avoidance (and other control objectives) in a multi-agent flocking scenario under the simplifying assumption of full state observation.

In [4], an approach based on Bayesian inference is proposed that allows an agent in a heterogeneous multi-agent environment to estimate the navigation model and goal of each of its neighbors. It then uses this information to compute a plan that minimizes inter-agent collisions while allowing the agent to reach its goal. Flocking formation is not considered.

### 7 Conclusions

With the introduction of Neural Flocking (NF), we have shown how machine learning in the form of Supervised Learning can bring many benefits to the flocking problem. As our experimental evaluation confirms, the symmetric and fully distributed neural controllers we derive in this manner are capable of achieving a multitude of flocking-oriented objectives, including flocking formation, inter-agent collision avoidance, obstacle avoidance, predator avoidance, and target seeking. Moreover, NF controllers exhibit real-time performance and generalize the behavior seen in the training data to achieve these objectives in a significantly broader range of scenarios.

Ongoing work aims to determine whether a DNC can perform as well as the centralized MPC controller for agent models that are significantly more realistic than our current point-based model. For this purpose, we are using transfer learning to train a DNC that can achieve acceptable performance on realistic quadrotor dynamics [1], starting from our current point-model-based DNC. This effort also involves extending our current DNC from 2-dimensional to 3-dimensional spatial coordinates. If successful, and preliminary results are encouraging, this line of research will demonstrate that DNCs are capable of achieving flocking with complex realistic dynamics.

For future work, we plan to investigate a distance-based notion of agent neighborhood as opposed to our current nearest-neighbors formulation. Furthermore, motivated by the quadrotor study of [21], we will seek to combine MPC with reinforcement learning in the framework of guided policy search as an alternative solution technique for the NF problem.
